# Rare Anatomic Variation: The Hepatosplenomesentericophrenic Trunk

**DOI:** 10.3390/medicina57020170

**Published:** 2021-02-15

**Authors:** Bogdan Gheorghe Hogea, Mugurel Constantin Rusu, Adelina Maria Jianu, Bogdan Adrian Manta, Adrian Cosmin Ilie

**Affiliations:** 1 Department of Orthopaedic & Trauma Surgery, Faculty of Medicine, “Victor Babes” University of Medicine and Pharmacy, 300041 Timisoara, Romania; hogeabg@yahoo.com; 2 Division of Anatomy, Faculty of Dental Medicine, “Carol Davila” University of Medicine and Pharmacy, 050474 Bucharest, Romania; mugurel.rusu@umfcd.ro (M.C.R.); bogdan.manta@drd.umfcd.ro (B.A.M.); 3 Department of Anatomy and Embryology, Faculty of Medicine, “Victor Babes” University of Medicine and Pharmacy, 300041 Timisoara, Romania; 4 Division of Public Health and Management, Faculty of Medicine, “Victor Babes” University of Medicine and Pharmacy, 300041 Timisoara, Romania; ilie.adrian@umft.ro

**Keywords:** celiac trunk, inferior phrenic artery, abdominal aorta, superior mesenteric artery, hepatic artery

## Abstract

The rare anatomic variants of the celiac trunk and superior mesenteric artery include the hepatosplenic, hepatosplenomesenteric (HSMT), celiacomesenteric, hepatomesenteric and gastrosplenic trunks. We report a 72-year-old female patient whose computed tomography angiograms indicated a rare anatomic feature whereby the right inferior phrenic artery was inserted in the origin of an HSMT, thus modifying it into a hepatosplenomesentericophrenic trunk (HSMPT). Above the HSMPT, the insertion of the left inferior phrenic artery in the origin of the left gastric artery determined a left gastrophrenic trunk (GPT). Proper identification of this type of rare anatomic variant is of utmost importance prior to different surgical procedures. For example, an HSMT origin of the right inferior phrenic artery is surgically relevant if this artery is an extrinsic pedicle of a hepatocellular carcinoma and is used for embolization of the tumor.

## 1. Introduction

The normal anterior (ventral) visceral branches of the abdominal aorta are the celiac trunk (CT), superior mesenteric artery (SMA) and inferior mesenteric artery (IMA). The normal anatomy of the CT indicates its trifurcation (*tripus Halleri*) in the common hepatic artery (CHA), left gastric artery (LGA) and splenic (or lienal) artery (SA) [1]. A true classical trifurcation occurs when all the three branches of the CT leave at the same point, while a false classical trifurcation occurs when the LGA leaves as a collateral branch, then the CT divides terminally into the SA and CHA. The normal anatomy of the CT can show a broad spectrum of anatomic variations [2,3].

According to Adachi (1928), quoted in [4,5], six types of arterial trunks can result after variable branching patterns of the CT: (1) hepatogastrosplenic trunk (86%), the basic variant; (2) hepatosplenic trunk (HST, 8%); (3) hepatosplenomesenteric trunk (HSMT, 1%); (4) celiacomesenteric trunk (1.5%); (5) hepatomesenteric trunk (0.5%) and (6) gastrosplenic trunk (3%). These types were found after Adachi documented 252 dissected cadavers. In Adachi’s third type, the HSMT, the CHA, SA, and SMA have a common origin, while the LGA arises directly from the aorta [4]. According to Lippert and Pabst [6], the HSMT occurs in 1% of cases [6]. The third type in Michels’ classification [7] also refers to a HSMT, but with a prevalence of 0.5%.

Song et al.’s definition of the common hepatic artery makes classification systems of variations in this region much easier [8]. These authors documented the celiac axis variations in 5002 patients [8]. They found HSMTs in 0.68% of those cases [8]. Aslaner et al. documented 1000 cases and found HSMTs in just 0.3% [9].

An HSMT can be associated with a gastrophrenic trunk (GPT), which, in turn, results from the common aortic origin of the inferior phrenic arteries and the LGA [4,10].

A different variant of HSMT was found associated with a hepatogastrophrenic trunk, with each trunk contributing roots for the CHA [11]. Only this last variant is documented as an HSMT in Bergman’s Comprehensive Encyclopedia of Human Anatomic Variation [12].

Here, we present an anatomic subvariant, that of a hepatosplenomesentericophrenic trunk (HSMPT), in which variant the right inferior phrenic artery origin was from an HSMT.

## 2. Anatomical Variation

During a retrospective study of archived computed tomography angiograms, the variant reported here was found in a 72-year-old female’s anonymized file. Informed consent for anonymous use of the data with scientific purposes was signed by the patient. The manuscript was tacitly approved by the responsible authorities where the work was carried out. The computed tomography examination used a previously detailed protocol [13,14]. It used a 32-slice scanner (Siemens Multislice Perspective Scanner, Erlangen, Germany) with a 0.6 mm collimation and reconstruction of 0.75 mm thickness. The arterial variant was documented using the Horos software.

**Figure 1:**
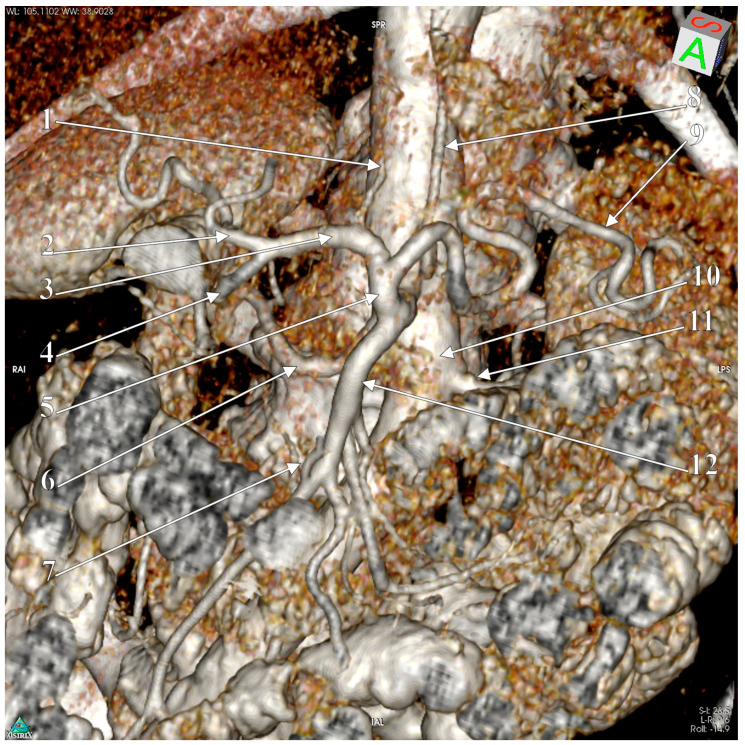
3D-VR of the hepatosplenomesentericophrenic trunk. 1. Right inferior phrenic a. (artery); 2. proper hepatic a.; 3. common hepatic a.; 4. gastroduodenal a.; 5. hepatosplenic trunk; 6. right renal a.; 7. middle colic a.; 8. left gastric a.; 9. splenic a.; 10. abdominal aorta; 11. left renal a.; 12. superior mesenteric a.

The slices showed an arterial trunk emerging from the aorta at the level of the first lumbar vertebra. It had a caliber of 5.86 mm and a length of 1.49 cm, and was directed antero-inferiorly. The left renal vein crossed the aorta anteriorly, inferior to that trunk. The right inferior phrenic artery departed from the origin of that trunk. The trunk was then divided into an upper, ascending HST, of 3.35 mm caliber, and a lower branch, the SMA, of 4.26 mm caliber. Therefore, that trunk was considered a hepatosplenomesentericophrenic trunk (HSMFT) and was documented by three-dimensional volume rendering (3D-VR) (Figure 1). The HST length was 1.31 cm, and it further divided into the SA and CHA. The CHA passed in front of the inferior cava vein and then passed anterior to the portal vein. It then divided into the proper hepatic and gastroduodenal arteries.

**Figure 2:**
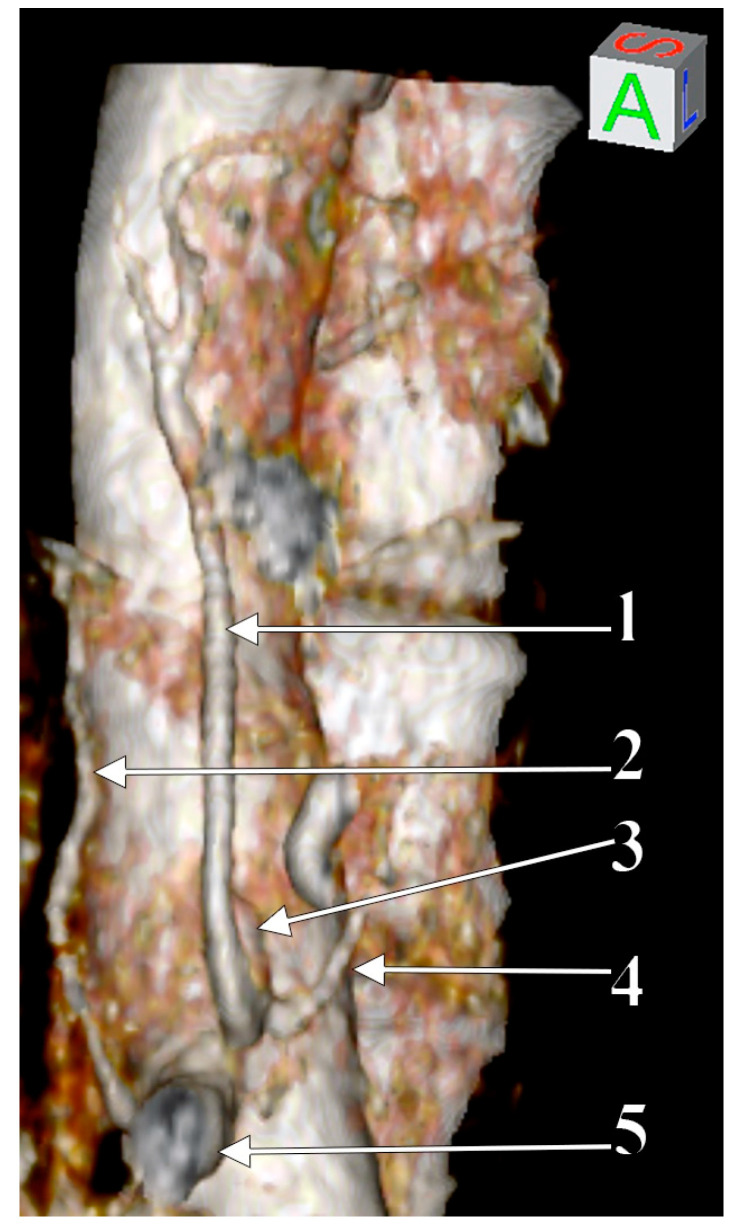
3D-VR of the reported arterial variant, depicting the inferior phrenic arteries. 1. Left gastric a.; 2. right inferior phrenic a.; 3. gastrophrenic trunk; 4. left inferior phrenic a.; 5. hepatosplenomesentericophrenic trunk.

The LGA and left inferior phrenic artery had a common aortic origin, thus forming a left gastrophrenic trunk (GPT) (Figure 2). The origin of the GPT was immediately superior to and left of the origin of the HSMPT. The origin of the HSMPT was at 9.37 mm superior to and left of the origin of the right renal artery.

## 3. Discussion

Accurate depiction of the CT and SMA anatomical features and variants in patients is of utmost importance due to the actual development of the interventional techniques for the surgical management of the liver [15], as well as of the neighboring organs. Knowledge of the anatomic possibilities of the celiaco-mesenteric axis is clinically important in liver transplantation; surgery of the liver, pancreas, esophagus and stomach; organ retrieval; and the treatment of abdominal aortic aneurysms [16].

The separate origin of the LGA from the HSMT could be explained by a developmental anomaly in which the separation of the Tandler’s longitudinal anastomosis of the ventral splanchnic arteries keeps the LGA with the first ventral segmental artery root. The next two roots then disappear, and the SA and CHA join the fourth root to form the HSMT [10,17].

**Figure 3:**
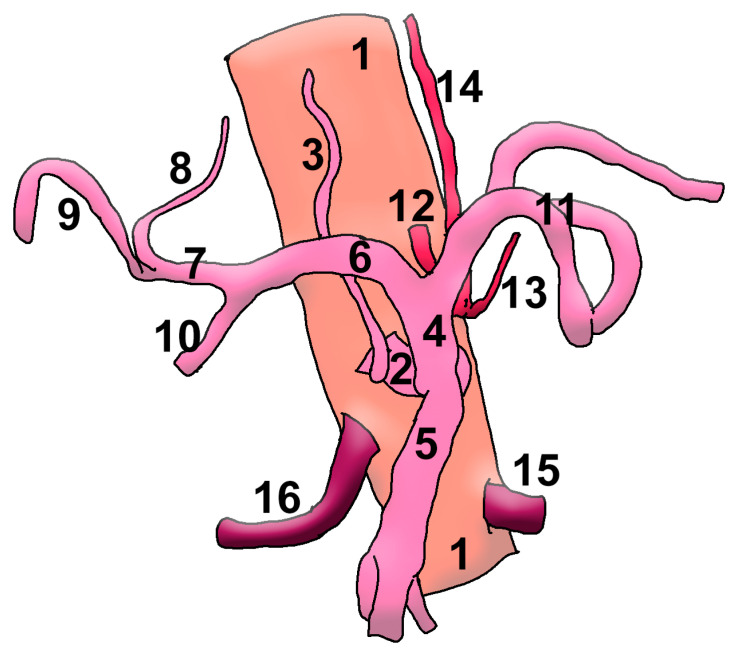
Drawing of the anatomic variant. 1. Aorta; 2. hepatosplenomesentericophrenic trunk; 3. right inferior phrenic a.; 4. hepatosplenic trunk; 5. superior mesenteric a.; 6. common hepatic a.; 7. proper hepatic a.; 8. left hepatic a.; 9. right hepatic a.; 10. gastroduodenal a.; 11. splenic a.; 12. gastrophrenic trunk; 13. left inferior phrenic a.; 14. left gastric a.; 15. left renal a.; 16. right renal a.

Adachi reported an HSMT in 1% of the 252 dissected cadavers [18], whereas other authors found it in 0.3% [9], 0.4% [19], 0.68% [8], 0.7% [20] or 1% [21]. To our knowledge, no other branch has been reported as arising from an HSMT. The right inferior phrenic artery (RIPA) has been reported to originate from various sources (e.g., celiac, aortic, left gastric, or renal artery) [9,22,23,24], but not from an HSMT, as in this case (Figure 3). A study on 300 cadavers found different origins of the right inferior phrenic artery, but no evidence was found for an SMA or HSMT origin [22]. Aslaner et al. documented the origins of the inferior phrenic arteries, separated or in common trunk, in 110 cases with celiac axis variation, with three of these cases being found with HSMTs [9]. However, in those cases the authors did not list any inferior phrenic arteries originating from the HSMT. A recent meta-analysis included 18 studies, which corresponded to 4208 patients, and found rare origins of the RIPA, with a pooled prevalence of 2.07% [24]. Among these were found 17 hepatic arteries, 2 SMAs and 1 dorsal pancreatic artery [24], but no HSMTs. Authors may report the prevalence of the inferior phrenic artery arising from the coeliac trunk but not consider the configuration of the coeliac trunk it is arising from. This may be a reason for such cases, like the subject of the current report, to be underreported.

The left inferior phrenic artery was found originating from the LGA in 2% [22], indicating the left GPT variant as rare. Different studies have reported GPTs, but these differed in origin, being either of aortic origin [4,25], as in our case, or of celiac origin [26]. Any inferior phrenic artery could combine with the LGA to form a GPT [26]. A common GPT results when the LGA combines with a common inferior phrenic trunk [4]. Different classifications do not perceive the GPT as a trunk [27]. A GPT is found associated either with an HSMT or an HST [27].

A previously reported variant [10] seems to have the closest morphology to the variant reported here. In that variant, which was also identified on CT angiograms, the inferior phrenic arteries arose from the LGA, and an HSMT was detected [10]. Another case has also been reported involving a left GPT and HSM combination; however, in that variant, the right inferior phrenic artery had an aortic origin [28]. The SMA origin of the right inferior phrenic artery has also been documented previously [29].

An accurate identification of the origin of the right inferior phrenic artery is important during the transcatheter embolization of an unresectable hepatocellular carcinoma [22], because the right inferior phrenic artery is most frequently involved in the supply and growth of this type of carcinoma [22]. Therefore, an HSMT origin of the right inferior phrenic artery should be added to the previously known possibilities (aortic, celiac, left gastric, renal or hepatic origin) [22]. Different authors disregard the inferior phrenic arteries when they pattern the CT [26]. Therefore, the prevalence of different celiac origins of the inferior phrenic arteries remains imprecise.

## 4. Conclusions

The rare variant presented here is, as previously concluded, of importance for anatomists, interventional radiologists and surgeons (vascular, abdominal or oncologic). The right inferior phrenic artery could be used for embolizations of hepatocellular carcinomas; therefore, a common origin of this artery with a rarely occurring HSMT, such as an HSMPT, should be kept in mind.
